# Prevalence and Risk Factors of Asymptomatic Colorectal Polyps in Taiwan

**DOI:** 10.1155/2014/985205

**Published:** 2014-06-23

**Authors:** Fu-Wei Wang, Ping-I Hsu, Hung-Yi Chuang, Ming-Shium Tu, Guang-Yuan Mar, Tai-Ming King, Jui-Ho Wang, Chao-Wen Hsu, Chiu-Hua Chang, Hui-Chun Chen

**Affiliations:** ^1^Department of Family Medicine, Kaohsiung Veterans General Hospital, Kaohsiung City 81362, Taiwan; ^2^Department of Public Health, Kaohsiung Medical University, Kaohsiung City 803, Taiwan; ^3^Division of Gastroenterology, Department of Internal Medicine, Kaohsiung Veterans General Hospital, Kaohsiung City 81362, Taiwan; ^4^Division of Cardiology, Department of Internal Medicine, Kaohsiung Veterans General Hospital, Kaohsiung City 81362, Taiwan; ^5^Division of Colorectal Surgery, Department of Surgery, Kaohsiung Veterans General Hospital, Kaohsiung City 81362, Taiwan; ^6^Department of Nursing, Kaohsiung Veterans General Hospital, Kaohsiung City 81362, Taiwan; ^7^Department of Radiation Oncology, Chang Gung Memorial Hospital, 123 TA-PEI Road, Kaohsiung City 833, Taiwan

## Abstract

*Purpose*. To investigate the prevalence and risk factors of hyperplastic and adenomatous colorectal polyps in a Taiwanese general population. *Methods*. From January 2009 to December 2011, consecutive asymptomatic subjects undergoing a routine health check-up were evaluated by colonoscopy. The colorectal polyps were assessed, and medical history and demographic data were obtained from each patient. Logistic regression analysis was conducted to search the independent risk factors for asymptomatic hyperplastic and adenomatous colorectal polyps. *Results*. Of the 1899 asymptomatic subjects, the prevalences of hyperplastic polyps and adenomatous polyps were 11.1% and 16.1%, respectively. Multivariate analysis revealed that high body mass index (BMI > 25: OR, 1.32, 95% CI, 1.05–1.71) and current smoking (OR, 1.87, 95% CI, 1.42–2.71) were independent predictors for hyperplastic colorectal polyps. Age over 60 years old (OR, 3.49, 95% CI, 1.86–6.51), high body mass index (BMI > 25: OR, 1.75, 95% CI, 1.21–2.71), heavy alcohol consumption (OR, 2.01, 95% CI, 1.02–3.99), and current smoking (OR, 1.31, 95% CI, 1.04–1.58) were independent predictors for adenomatous colorectal polyps. *Conclusion*. High BMI and smoking are common risk factors for both adenomatous and hyperplastic polyps. Old age and alcohol consumption are additional risk factors for the development of adenomatous polyps.

## 1. Introduction

Colorectal cancer is one of the most common cancers worldwide. In Taiwan, the incidence of colorectal cancer is increasing as lifestyles and diets have become more westernized. In the United States, by contrast, the incidence of colorectal cancer is declining probably because of advances in cancer screening. Many studies suggested most colorectal cancers originate from a precursor benign polyp [1, 2], which makes this cancer potentially preventable by appropriate screening colonoscopy programs in patients at increased risk. Evidence shows that screening asymptomatic populations beginning at age 50 years can reduce mortality due to colorectal cancer [3, 4] and that removal of precursor adenomas may reduce the incidence of colorectal cancer [5]. Identification of important risk factors for colorectal neoplasia could inform both risk stratification and development of risk reduction strategies.

Until approximately 1990, colorectal polyps were classified into two groups: adenomatous polyps (conventional adenoma) and hyperplastic polyps. Conventional adenomas are precursor lesions to colorectal carcinomas developing via the traditional adenoma-carcinoma pathway characterized by chromosomal instability [6]. Many risk factors for colorectal adenomatous polyps in the Western countries have been well identified previously, such as current smoking, moderate to heavy alcohol use, metabolic syndrome, and lack of physical activity [7–11]. However, only a few studies have been conducted to investigate the risk factors for colorectal polyps in Asian countries. Recently, a cross-sectional study from Korea showed an association between colorectal adenoma and abdominal obesity [12], but a prospective autopsy study in Hawaii Japanese subjects showed no associations between colorectal adenomatous polyps and intake of dietary fat, proteins, or carbohydrates and body mass index, level of physical activity, and cigarette smoking history [13].

Hyperplastic polyps are traditionally assumed to have no malignant potential. However, recent studies showed approximately 30% of colorectal carcinomas develop via the serrated neoplasia pathway characterized by frequent BRAF mutation and widespread DNA methylation [14]. Most national guidelines for colonoscopy surveillance after polypectomy agree that patients with small, distally located hyperplastic polyps do not require subsequent surveillance [15]. Nonetheless, it is recommended that patients with proximally located hyperplastic polyps or hyperplastic polyps greater than 5 mm are followed up at 5 years [14, 15].

Currently, the risk factors for developing hyperplastic polyps remain unclear, and the results of risk factors for adenomatous polyps in Asian countries are quite conflicting. We therefore conducted this cross-sectional study to investigate the risk factors for the development of colorectal hyperplastic polyps and adenomatous polyps in Taiwan.

## 2. Methods

### 2.1. Subjects

From January 2009 to December 2011, consecutive asymptomatic subjects undergoing a routine colonoscopy during health check-up were invited to participate in this study. The study protocol was approved by institutional review boards at Kaohsiung Veterans General Hospital, and all participants provided written informed consent. The eligible subjects were excluded if they reported symptoms of lower gastrointestinal tract disease, including rectal bleeding, marked change in bowel habits, or lower abdominal pain that would normally require medical evaluation. Other exclusion criteria were current participation in other studies, history of disease of the colon (such as colitis, polyps, or cancer), prior colonic surgery, and colorectal examination (i.e., sigmoidoscopy, colonoscopy, or barium enema) within the previous 10 years.

### 2.2. Study Design

A complete history and physical examination were performed for every subject undergoing health check-up. All subjects were carefully queried regarding the presence of abdomen symptoms in the previous 1 month, and subjects who responded negatively were classified as asymptomatic subjects and enrolled for this study. All the participants received anthropometric and blood biochemical tests, which included fasting plasma glucose, serum triglyceride, and high-density lipoprotein (HDL) cholesterol level, and received total colonoscopy. Colonoscopies were performed by three experienced endoscopists (King TM, Wang JH, and Hsu CW) with the Olympus PCF-Q240AL and PCF-Q260AL (Olympus Corp., Tokyo, Japan) after the subjects had fasted overnight. Bowel preparation was performed with Fleet oral saline laxative using the same protocol as that used for diagnostic colonoscopy. The patients were carefully examined for colorectal mucosal lesion. Colorectal polyp was defined as a protuberance into the lumen from the normally flat colonic mucosa. The location of the colorectal polyp was divided into the proximal colon, including the cecum, the ascending colon and transverse colon, and the distal colon, including the splenic flexure, the descending colon, the sigmoid colon, and the rectum. All visible polyps were removed and examined histologically by the pathologist. The pathology types of colorectal polyps were subsequently categorized into hyperplastic polyps, adenomatous polyps, sessile serrated adenoma, and traditional serrated adenoma.

To assess the relations between clinical characteristics and asymptomatic colorectal polyps, the following data were recorded for each subject: age; gender; educational status; family history of colorectal polyps; consumption of tobacco, alcohol, coffee, tea, spicy foods, or betel nut; exercise habit; whether vegetarian or not; and longtime use of nonsteroidal anti-inflammatory drugs (NSAIDs). All variables were categorized for data analyses.

### 2.3. Statistical Analysis

The chi-square test or Fisher's exact test was employed to investigate the relationships between the rate of colorectal polyps and clinical characteristics. These variables included the following: gender; age (<45, 45–60, or >60 years); educational status (<10, 10–12, or >12 years); body mass index (BMI: <25, 25–30, or >30); NSAID use (yes or no); family history of colorectal polyps (yes or no); smoking status (no, former smoking, or current smoking); consumption of alcohol, coffee, tea, or spicy foods; exercise habit (no, ≤3 times per week, or >3 times per week); and betel nut habit and vegetarian (yes or no). Metabolic syndrome was defined according to the modified National Cholesterol Education Program Adult Treatment Panel III definition for South Asians and Chinese. A *P* value less than 0.05 was considered significant. Significant variables revealed by univariate analysis were subsequently assessed by a stepwise logistic regression method to identify independent clinical factors predicting the presence of colorectal polyps in asymptomatic subjects.

All statistical analyses were performed using SPSS version 17.0 (SPSS Inc. Chicago, II, USA).

## 3. Results

### 3.1. Patient Demographics and Colonoscopic Characteristics

From January 2009 to December 2011, 1899 asymptomatic subjects (mean age, 52.8 ± 10.6 years; age range, 16–86 years; male/female, 1203/696) were recruited for this study. Among them, 520 (27.4%) had colorectal polyps (Table 1). The prevalences of hyperplastic polyps, adenomatous polyps, sessile serrated adenoma, and traditional serrated adenoma were 11.1%, 16.1%, 1.8%, and 0.7%, respectively.

### 3.2. Risk Factors for the Development of Colorectal Hyperplastic Polyps

Table 2 shows the results of univariate analysis for the risk factors for the development of colorectal hyperplastic polyps. Current smoking, old age, and higher BMI were significantly associated with hyperplastic polyps formation (*P* < 0.001, 0.048, and 0.004, resp.). The subjects with and without hyperplastic polyps had comparable gender, education level, family history of colorectal polyps, alcohol, coffee, tea, and spicy food consumption, betel nut use, exercise habit, vegetarian, metabolic syndrome status, and NSAID use. Multivariate analysis with stepwise logistic regression showed that both current smoking (OR, 1.87, 95% CI, 1.42–2.71) and high BMI (BMI > 25: OR, 1.32, 95% CI, 1.05–1.71) were independent predictors for asymptomatic hyperplastic colorectal polyps (Table 3).

### 3.3. Risk Factors for the Development of Colorectal Adenomatous Polyps

Table 4 displays the results of univariate analysis for the risk factors for developing colorectal adenomatous polyps. The subjects with adenomatous polyps were less educated than those without adenomatous polyps. Additionally, old age, male gender, current smoking, and heavy alcoholic drinking habit were significantly higher in the adenomatous polyps group (*P* < 0.001, 0.017, 0.011, and 0.005, resp.). Further, the subjects with adenomatous polyps had higher BMI than those without adenomatous polyps. However, the two groups had comparable family history of colorectal polyps, coffee, tea, and spicy food consumption, betel nut use, exercise habit, vegetarian, metabolic syndrome status, and NSAID use.

Multivariate analysis revealed that age over 60 years old (OR, 3.49, 95% CI, 1.86–6.51), high BMI (BMI > 25: OR, 1.75, 95% CI, 1.21–2.71), heavy alcohol consumption (OR, 2.01, 95% CI, 1.02–3.99), and current smoking (OR, 1.31, 95% CI, 1.04–1.58) were independent predictors for asymptomatic adenomatous polyps (Table 5).

## 4. Discussion

The current study demonstrated that the prevalences of colorectal hyperplastic polyps and adenomatous polyps in asymptomatic Taiwanese were 11.1% and 16.1%, respectively. Smoking and high BMI are risk factors for both adenomatous and hyperplastic polyps. Old age and alcohol consumption are additional risk factors for the development of adenomatous polyps. To our knowledge, this is the first work simultaneously investigating the risk factors for adenomatous and hyperplastic polyps in an asymptomatic Asian population.

In this study, smoking is independent risk factor for developing both adenomatous and hyperplastic polyps with odds ratios between 1.31 and 1.87, respectively. In previous studies, cigarette smoking has consistently been a risk factor for colorectal adenoma [16]. Nonetheless, a Minnesota colonoscopy-based study also found that smoking was strongly associated with increased risk of hyperplastic polyps [17]. Several known or probable human carcinogens are present in cigarette smoke, including polycyclic amines, aromatic amines, and benzene [18]. Martínez et al. reported that APC and KRAS mutations were found in 36% and 61% of the hyperplastic polyps of smokers but were absent in nonsmokers [19]. Recent studies also demonstrated that smoking is associated with DNA hypermethylation, which has been implicated in the pathogenesis of hyperplastic polyp [20].

This study revealed a strong positive association between BMI and both adenomatous and hyperplastic colorectal polyps. Previous studies indicate a higher BMI considered as overweight or obesity has revealed associations with the risk of colorectal adenomas and hyperplastic polyps [21, 22], but not all investigations [23]. Higher BMI levels have been more strongly associated with advanced lesions than with nonadvanced, tubular adenomas [24]. In a prospective study in which large bowel adenomas were observed over a 3-year period, the authors reported a positive association between obesity and adenoma growth [25]. The mechanism by which obesity increases the risk of asymptomatic colorectal polyps is unknown. Possible explanations include the inflammation, oxidative stress, and insulin resistance in obese subjects [26, 27].

Our study also showed that old age and heavy alcohol consumption were independent predictors for developing adenomatous colorectal polyps in asymptomatic subjects. Advanced age is a well-known risk factor for the development of colorectal adenomas and advanced neoplasm [28]. Colonoscopic screening studies in asymptomatic people suggest that the prevalence of adenomas is about 25 to 30 percent at age 50 [29, 30]. Autopsy studies have found rates as high as 50 percent by age 70 [31], compared with only 1 to 4 percent in those in their twenties or thirties [32]. In this study, age over 60 years old was independent predictor for developing asymptomatic colorectal adenomatous polyp whereas it was not a risk for the development of hyperplastic polyp. The findings were supported by a previous study [10].

Regular alcohol intake has been reported as a risk factor for both colorectal adenoma and cancer in some but not all previous studies [33, 34]. Very few studies have evaluated the association of hyperplastic polyp risk with alcohol intake, and only one of these studies also considered the combination of hyperplastic and adenomatous polyps [35]. The current cross-sectional study revealed consumption of at least three times alcoholic drinks per week was independent factor predicting the development of colorectal adenomatous polyps in asymptomatic subjects. The mechanism by which alcohol may affect polyp risk is not known. Some of the proposed mechanisms are nutritional deficiency, including folate deficiency, effects of acetaldehyde, such as hyperregeneration of colonic mucosa, activation of procarcinogens, and generation of reactive oxygen species through the induction of cytochrome P-450 2E1, abnormal DNA methylation, and immune system suppression [36].

Regarding serrated adenomas, the prevalences of sessile serrated adenoma and traditional serrated adenoma in the current study were 1.8% and 0.7%, respectively. Compared with previous literatures [37, 38], the prevalences of sessile serrated adenoma and traditional serrated adenoma in this study were relatively low. The variable prevalences of serrated adenomas in different countries can be explained by ethnic difference, individual endoscopist's detection rate, pathologist interpretation, and differences in the selected populations.

Despite its contributions, this study had certain limitations. First, self-selection bias of the population in this trial was possible because all enrolled subjects had undergone self-paid health examination and likely had better economic status than the general population in Taiwan (Table 1). Second, the studied subjects may differ from the subjects in primary care hospital because our hospital is a tertiary care center.

## 5. Conclusion

In conclusion, the prevalence of colorectal polyps in asymptomatic subjects is 27.4% in Taiwan. High BMI and smoking are common risk factors for both adenomatous and hyperplastic polyps. Advanced age and alcohol consumption are additional risk factors for the development of adenomatous polyps of colorectum. The findings suggest that lifestyle modification including adequate control of body weight and abstaining from smoking and alcohol may potentially decrease the risk of developing colorectal adenomatous polyps. Further studies are warranted to investigate the mechanisms underlying the association of these lifestyle factors with the risk of adenomatous and hyperplastic polyps of the colorectum and to elucidate whether abstaining from smoking and alcohol really can reduce the incidence of recurrent colorectal polyps or advanced neoplasia.

## Figures and Tables

**Table 1:** Demographics and endoscopic findings of asymptomatic health check-up subjects (*n* = 1899).

Clinical characteristics	
Age, *n* (%)	
Mean (SD) yr.	52.8 (10.6)
<45 yr.	393 (20.7)
45–60 yr.	1038 (54.7)
>60 yr.	468 (24.6)
Height, cm	165.8 (8.2)
Weight, kg	66.2 (12.3)
Gender, *n* (%)	
Men	1203 (63.3)
Women	696 (36.7)
Body mass index, *n* (%)	
Mean (SD)	23.9 (3.4)
<25	1233 (64.9)
25–30	573 (30.2)
>30	93 (4.9)
Educational level, *n* (%)	
Middle school	144 (7.6)
High school	799 (42.1)
University	641 (33.7)
Graduate school	315 (16.6)
Yearly salary (US dollar), *n* (%)	
Less than 10,000	182 (9.6)
From 10,000 to 30,000	984 (51.8)
More than 30,000	733 (38.6)
Colonoscopic findings, *n* (%)	
Normal	1379 (72.6)
Colorectal polyps	520 (27.4)
Pathological type	
Hyperplastic polyps	210 (11.1)
Adenomatous polyps	305 (16.1)
Sessile serrated adenoma	34 (1.8)
Traditional serrated adenoma	14 (0.7)
Location	
Proximal colon only	99 (5.2)
Distal colon only	343 (18.1)
Both	78 (4.1)

**Table 2:** Univariate analysis of the risk factors for the development of colorectal hyperplastic polyps.

Principal parameter	Hyperplastic polyp (−)	Hyperplastic polyp (+)	*P* value
Sex, *n* (%)			0.195
Men	837 (60.7)	139 (66.1)	
Women	542 (39.3)	71 (33.9)	
Age (yr.), *n* (%)			0.048
<45	318 (23.1)	40 (19.2)	
45–60	778 (56.4)	114 (54.1)	
>60	283 (20.5)	56 (26.7)	
Education (yr.), *n* (%)			0.136
<10	301 (21.8)	43 (20.6)	
10–12	539 (39.1)	92 (44.0)	
>12	539 (39.1)	75 (35.4)	
BMI∗, *n* (%)			0.004
<25	949 (68.8)	114 (54.2)	
25–30	363 (26.3)	83 (39.4)	
>30	67 (4.9)	13 (6.4)	
NSAID^†^ use, *n* (%)			0.836
No	1329 (96.4)	201 (95.9)	
Yes	50 (3.6)	9 (4.1)	
Family history of colon polyp, *n* (%)			0.368
No	1208 (87.6)	178 (84.9)	
Yes	171 (12.4)	32 (15.1)	
Smoking status, *n* (%)			<0.001
Never smoking	896 (65.0)	112 (53.3)	
Former smoking	200 (14.5)	25 (11.9)	
Current smoking	283 (20.5)	73 (34.8)	
Alcohol drinking, *n* (%)			0.186
No	1019 (73.9)	148 (70.6)	
≦3 times per week	283 (20.5)	45 (21.6)	
>3 times per week	77 (5.6)	17 (7.8)	
Coffee drinking, *n* (%)			0.924
No	749 (54.3)	122 (58.2)	
≦3 times per week	262 (19.0)	38 (17.9)	
>3 times per week	368 (26.7)	50 (23.9)	
Tea drinking, *n* (%)			0.481
No	513 (37.2)	89 (42.2)	
≦3 times per week	298 (21.6)	38 (17.9)	
>3 times per week	568 (41.2)	83 (39.9)	
Spicy foods consumption, *n* (%)			0.079
No	778 (56.4)	110 (52.3)	
≦3 times per week	412 (29.9)	55 (26.4)	
>3 times per week	189 (13.7)	45 (21.3)	
Betel nut use, *n* (%)			0.728
No	1294 (93.8)	198 (94.5)	
Yes	85 (6.2)	12 (5.5)	
Exercise habit, *n* (%)			0.867
No	469 (34.0)	70 (33.5)	
≦3 times per week	389 (28.2)	63 (29.8)	
>3 times per week	521 (37.8)	77 (36.7)	
Vegetarian, *n* (%)			0.675
No	1335 (96.8)	202 (96.3)	
Yes	44 (3.2)	8 (3.7)	
Metabolic syndrome, *n* (%)			0.078
No	1019 (73.9)	140 (66.9)	
Yes	360 (26.1)	70 (33.1)	

*BMI = body mass index, indicating weight in kg divided by body surface area. ^†^NSAID: nonsteroid anti-inflammatory drug.

**Table 3:** Independent risk factors for the development of colorectal hyperplastic polyp.

Clinical variable	Coefficient	SE	OR (95% CI^†^)	*P* value
BMI^‡^ > 25	0.373	0.284	1.32 (1.05–1.71)	0.017
Current smoking	0.672	0.359	1.87 (1.42–2.71)	0.009

^†^CI = confidence interval.

^‡^BMI = body mass index, indicating weight in kg divided by body surface area (body mass index).

**Table 4:** Univariate analysis of the risk factors for the development of colorectal adenomatous polyps.

Principal parameter	Adenomatous polyp (−)	Adenomatous polyp (+)	*P* value
Sex, *n* (%)			0.017
Men	837 (60.7)	219 (71.9)	
Women	542 (39.3)	86 (28.1)	
Age (yr.), *n* (%)			<0.001
<45	319 (23.1)	39 (12.9)	
45–60	778 (56.4)	153 (50.0)	
>60	282 (20.5)	113 (37.1)	
Education (yr.), *n* (%)			0.015
<10	301 (21.8)	72 (23.7)	
10–12	539 (39.1)	150 (49.2)	
>12	539 (39.1)	83 (27.1)	
BMI∗, *n* (%)			0.001
<25	949 (68.8)	161 (52.7)	
25–30	363 (26.3)	131 (42.9)	
>30	67 (4.9)	13 (4.4)	
NSAID^†^ use, *n* (%)			0.875
No	1329 (96.4)	293 (96.1)	
Yes	50 (3.6)	12 (3.9)	
Family history of colon polyp, *n* (%)			0.603
No	1208 (87.6)	264 (86.4)	
Yes	171 (12.4)	41 (13.6)	
Smoking status, *n* (%)			0.011
Never smoking	896 (65.0)	171 (56.2)	
Former smoking	200 (14.5)	41 (13.5)	
Current smoking	283 (20.5)	93 (30.3)	
Alcohol drinking, *n* (%)			0.005
No	1019 (73.9)	214 (70.2)	
≦3 times per week	283 (20.5)	50 (16.4)	
>3 times per week	77 (5.6)	41 (13.4)	
Coffee drinking, *n* (%)			0.126
No	749 (54.3)	177 (58.0)	
≦3 times per week	262 (19.0)	59 (19.5)	
>3 times per week	368 (26.7)	69 (22.5)	
Tea drinking, *n* (%)			0.557
No	513 (37.2)	122 (40.1)	
≦3 times per week	298 (21.6)	54 (17.6)	
>3 times per week	568 (41.2)	129 (42.3)	
Spicy food consumption, *n* (%)			0.123
No	778 (56.4)	177 (58.0)	
≦3 times per week	412 (29.9)	72 (23.7)	
>3 times per week	189 (13.7)	56 (18.3)	
Betel nut use, *n* (%)			0.725
No	1293 (93.8)	284 (93.1)	
Yes	86 (6.2)	21 (6.9)	
Exercise habit, *n* (%)			0.694
No	469 (34.0)	111 (36.4)	
≦3 times per week	389 (28.2)	86 (28.1)	
>3 times per week	521 (37.8)	108 (35.5)	
Vegetarian, *n* (%)			0.543
No	13358 (96.8)	298 (97.7)	
Yes	44 (3.2)	7 (2.3)	
Metabolic syndrome, *n* (%)			0.168
No	1019 (73.9)	213 (69.8)	
Yes	360 (26.1)	92 (30.2)	

*BMI = body mass index, indicating weight in kg divided by body surface area. ^†^NSAID: nonsteroid anti-inflammatory drug.

**Table 5:** Independent risk factors for the development of colorectal adenomatous polyp.

Clinical variable	Coefficient	SE	OR (95% CI^†^)	*P* value
Age > 60 y/o	1.352	0.362	3.49 (1.86–6.51)	<0.001
Alcohol drinking > 3 times per week	0.545	0.358	2.01 (1.02–3.99)	0.007
BMI^‡^ > 25	0.634	0.245	1.75 (1.21–2.71)	0.006
Current smoking	0.318	0.273	1.31 (1.04–1.58)	0.023

^†^CI = confidence interval.

^‡^BMI = body mass index, indicating weight in kg divided by body surface area (body mass index).
